# Chloroform Administration in the Midlands

**Published:** 1898-12-24

**Authors:** 


### CHLOROFORM ADMINISTRATION IN THE MIDLANDS.

Dr. W. J. McCardie, anæsthetist to the General Hospital, Birmingham, describes[1] a method of giving chloroform which is, he says, very largely used in the Midlands, more especially in gynæcological work. It consists of the administration of a mixture of one part of chloroform and two parts of ether, by measure, in a Clover's smaller inhaler without the bag. The results seem to have been good. One naturally feels inclined to ask wherein lies the advantage of this mixture, and, of course, one is answered at once by the assertion that the stimulating effect of the ether counteracts the depressing influence of the chloroform. Perhaps. But we fancy that there are other influences at work which make for safety, although they may hardly tend much towards efficiency, in the method described. What is obvious is that whatever the quantity of the mixture in the inhaler the evaporating surface is strictly limited, that in consequence of the admixture of ether the temperature at which the evaporation takes place is kept very low and thus that the amount of vapour which can be inhaled at each respiration will be kept within a moderate amount. Hence probably the safety. On the other hand, hence also the difficulties which, admittedly, sometimes accompany the process. Although a mixture of two anæsthetics is employed in this method it must be remembered that it is really a means of obtaining anæsthesia by chloroform, not by ether; and that beyond the stimulating effect of the ether this drug probably acts mainly as an automatic check to the amount of chloroform given off. The construction of the inhaler also enables one to regulate with great nicety the proportion between the fresh and the vapour-charged air, so that the method described is clearly one which is working in the right direction, *i.e.*, in the direction of giving the administrator control over the amount of vapour which is inhaled. Whether this is the best way of getting such control is another question.

